# Tarsal Coalitions: A Practical Approach to a Not-So-Rare Entity

**DOI:** 10.5334/jbr-btr.1224

**Published:** 2016-11-19

**Authors:** Christian Glaser

**Affiliations:** 1 Institute of Clinical Radiology, LMU Grosshadern, NYUMC, USA, DE

**Keywords:** Tarsal coalition, talocalcaneal, calcaneonavicular, ankle, hindfoot

The term *coalition* refers to a connection between two – according to standard anatomical definitions – normally separate bones, presumably due to a disturbance in mesenchymal segmentation. A coalition may be bony, fibrous, cartilaginous, or a mixture of these tissues and may be partial or complete, i.e. affect a complete joint (facet) surface area or a fraction of it.

*Segmentation* refers to a process in the unborn when the future bones’ centers start to separate out of a common cartilaginous plate or volume in the foot. This process is thought to occur by constrictions progressing from the periphery towards the center of the common bone plate until a new bone center is separated out of the common plate with the trace of the constrictions delineating the future joint space. It is hypothesized that the time and extent that this process is disturbed or interrupted determines the type of coalition: early disturbance will result in a partial or complete bony coalition; disturbance later in the process of segmentation will result in a fibrous, fibrocartilaginous, or cartilaginous partial or complete coalition.

While originally the incidence of tarsal coalitions was thought to be around 1%, more recently, it is estimated to be up to 13%. Coalitions may occur bilaterally in up to 60–70% and two coalitions in the same foot are described in 10–15%. This is clinically relevant if a surgical resection/correction of a coalition is considered. Inheritance is considered unifactorial, autosomal dominant with high penetrance. The most common forms of coalitions are the talocalcaneal – especially of the middle subtalar joint facet – and calcaneonavicular forms. Talonavicular and calcaneocuboidal or tarsometatarsal coalitions are less common.

**Figure 1:**
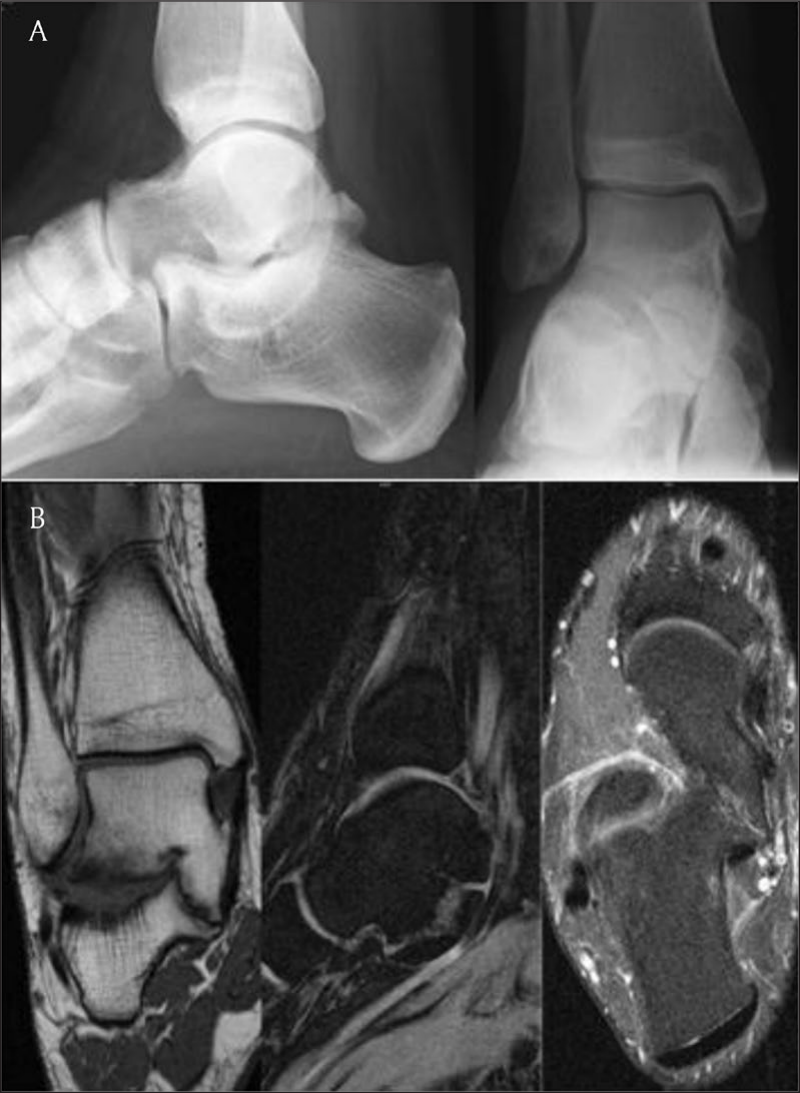
A 23-year-old male patient complains about obtuse pain in the right hindfoot initiating spontaneously, without a history of trauma and increasing under loading since one year. **(a)** Standard radiographs show no evidence of OA, inflammatory disease or bone destruction. But one can appreciate a C-sign (i.e. there is an apparent continuity in the bony contour of the talar dome to the sustentaculum tali) and the sustentaculum tali does not show the typical ‘brick’ shape but appears somehow roundish in the lateral view. Note the small bony prominence somewhat posterior to the margin of the talonavicular joint, consistent with a subtle talar beaking. In the frontal view, note the abnormal angulation of the middle talocalcaneal joint facet from the coronal towards the sagittal plane. **(b)** MRI (from left to right: T1w cor, Flash 3D Water Excitation sag, moderately T2w FS ax) reveals a fibrocartilaginous coalition of the middle talocalcaneal joint facet and demonstrates the abnormal angulation of the joint facet to more detail. There is but subtle periarticular sclerosis and bone marrow edema pattern.

Coalitions in one group of patients tend to become symptomatic, i.e. painful, with progressive ossification of the normal bones as well as of potential coalitions, with less general intrinsic flexibility (ossifying skeleton) as well as locally restricted movement (coalition) in the foot. Hence the age of presentation mostly is in adolescence. Following the age pattern of foot ossification, calcaneonavicular coalitions tend more to become symptomatic in the earlier teen years; talocalcaneal ones become symptomatic more towards the later teen years [1]. Yet, another subset of patients remain symptom-free until an increase in physical activity or a traumatic event, e.g. an ankle sprain, occur – ‘awakening’ a formerly asymptomatic coalition. Often, a calcaneonavicular coalition is symptomatic at the location of the coalition, whereas a talocalcaneal coalition may present not infrequently with a but poorly localized pain ‘somewhere deep’ in the hindfoot (Figure 1).

Although the amount of foot deformity varies considerably within and between any type of tarsal coalitions, one frequent and central pathomechanism is (direct or indirect) limitation of subtalar joint movement, a condition that considerably increases stress on the adjacent joints and that may ultimately result in a flatfoot configuration [2]. Excessive peroneal tension and secondary shortening of the peroneal tendons due to restricted subtalar inversion are frequent. Hence, pain in coalitions may also arise in other locations than the coalition itself.

Therapeutic strategies include restricting any physical activities that seem to provoke the symptoms; also orthoses, temporary cast immobilization, especially in children, may significantly improve the condition. NSAID can contribute to relief secondary inflammatory changes and pain. Talocalcaneal coalitions appear to respond better to conservative treatment options than to other forms. Surgical treatment after unsuccessful conservative therapy can be excision of the coalition (technically easier in calcaneonavicular coaltions and benefitting from the high regenerative potential in young patients) – in the absence of secondary degenerative change – and/or arthrodesis of the involved joints can be performed, sometimes guided by diagnostic injections of local anesthetics to determine the (most) painful joints.

**Figure 2:**
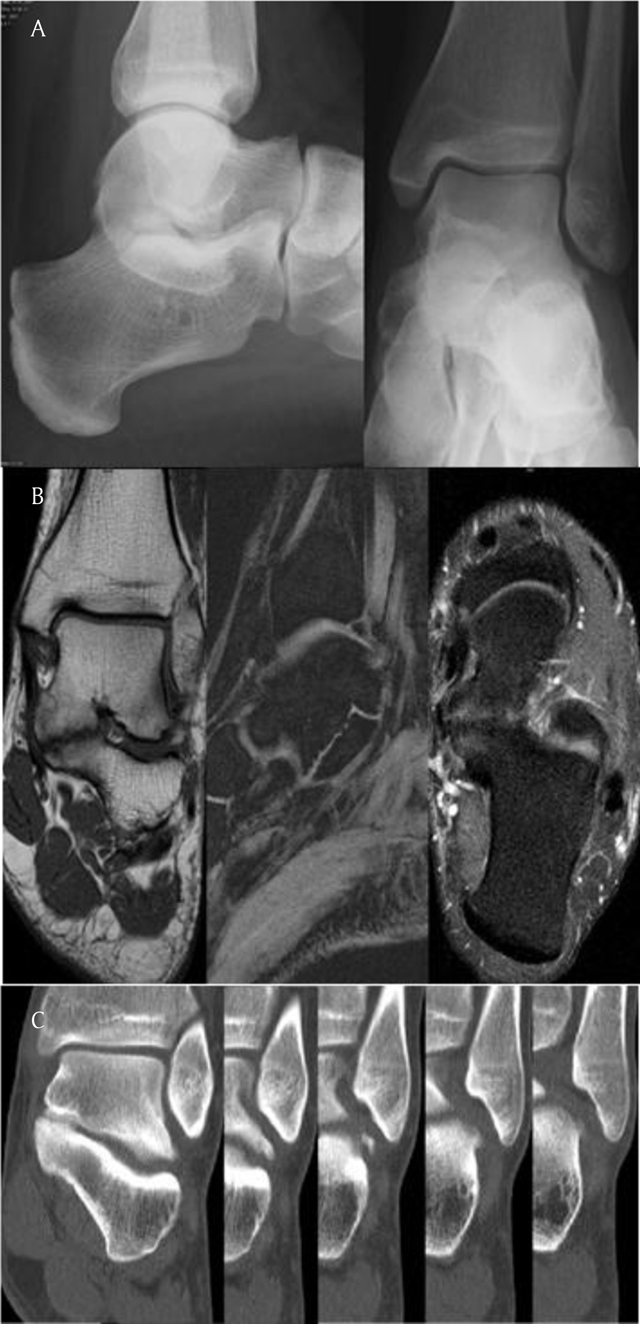
One year later, the same patient complains about newly arisen pain in the left hindfoot. **(a)** In the lateral view there is an ‘incomplete C-sign’, the sustentaculum is almost in perfect ‘brick’ shape. **(b)** MRI shows another coalition of the middle talocalcaneal facet. The MRI signal characteristics are in favor of a strong cartilaginous and less fibrous components and two subtle almost complete bony bridges across the joint space. Note that the orientation of the facet is less angulated out of its normal anatomic plane than in the right foot but there is more bone marrow edema pattern around the joint. **(c)** In this same patient’s left hindfoot, CT makes us appreciate an additional small bony bridge at the lateral aspect of the posterior talocalcaneal joint facet, consistent with an ‘extraarticular coalition’. This is a finding that should not be missed if surgery is considered.

Imaging should be based on radiographs followed by MRI and – if relevant for therapy – by CT (Figure 2). The role of imaging is to corroborate the suspicion of a coalition, to bring up a coalition as a potential differential diagnosis to explain unclear or prolonged pain, to determine the nature, extent, and location of one or more coalitions as well as potential accompanying changes (degenerative or stress reactions, e.g. bone marrow edema pattern) in order to help guide therapy. X-rays should comprise a lateral view of the ankle/lateral weight-bearing view including hindfoot and midfoot and an oblique 45° projection of the hindfoot and midfoot. Depending on the initial clinical setting, often a frontal ankle view and/or a dorsoplantar projection of the foot may be available. Here are some imaging findings indicating a potential coalition [3].

*Talar beaking* is a bony prominence or spur of variable extent at the distal dorsal aspect of the talus attributed to increased traction on the dorsal talonavicular ligament and its periosteal insertion following restricted motion in the subtalar joints. This finding must not be confounded with an osteophyte in the talonavicular joint, which would be located at the joint surface/margin and be an argument against a potential surgical procedure.

The *anteater sign*, an extension of the prominent anterior process of the calcaneus towards the lateral aspect of the navicular, indicative of a calcaneonavicular coalition is well seen in the oblique view. Similarly, a bony prominence on the lateral aspect of the navicular towards the anterior process of the calcaneus may be present, the so-called *reverse anteater sign*. In the case of a complete bony calcaneonavicular coalition, that bony bridge may present as the *bony bar sign*.

The *absent middle facet sign* relates to an ill-defined or completely obscured middle subtalar joint facet in well-obtained lateral radiographs. The *C-sign* is a C-shaped projected contour in complete or incomplete continuity from the sustentaculum tali to the posterior projection of the talus in the lateral view. Finally, the *dysmorphic sustentaculum* describes a roundish and broad projection of the sustentaculum tali as opposed to its regular flat rectangular aspect in the lateral view resulting from the dysmorphic and abnormally tilted middle subtalar joint. The three latter findings may indicate a talocalcaneal coalition.

The technical evolution of MRI enables this modality to depict and accurately describe any coalition and its morphologic features, reducing the need for CT in the work-up of coalitions. Both CT and MRI have greatly facilitated the diagnosis of tarsal coalitions compared to radiography; this is also reflected by the higher incidence of coalitions in cross-sectional imaging studies. MRI shows *bone marrow edema pattern* around a non-osseous coalition or around adjacent joints; this can be used for the detection of coalitions and illustrates some of the mechanical impairment and stress brought upon the foot by a coalition. And of course, MRI may demonstrate associated soft-tissue changes such as tendon pathologies in foot deformity or sinus tarsi syndrome. In terms of MRI analysis, it is helpful to systematically look for the presence or absence/disturbance of the *typical sequence of linear patterns reflecting a joint*: trabecular bone, subchondral bone plate, articular cartilage, joint space, articular cartilage, subchondral bone plate, trabecular bone. This approach may help to avoid overlooking subtle coalitions.

In specific situations, such as pre-operatively, CT is useful in deciding whether a resection or arthrodesis is feasible, specifically planning a procedure, assessing the fine bony details of a coalition, or to assessing secondary subtle degenerative change [4]. It may also be especially helpful to detect small bony bridges outside/at the very periphery of the main joints of the foot, so-called extra-articular coalitions.
